# Book Reviews

**Published:** 1902-04

**Authors:** 


GYNECOLOGICAL PATHOLOGY. A Manual of Microscopic Technique and Diagnosis in Gynecological Practice. For Students and Physicians. By DR. CARL ABEL, Privat-Docent, Berlin. Translated and edited by Samuel Wyllis Baudler, M. D., Adjunct Gynecologist to the Beth Israel Hospital, New York. With a chapter on the embryology of the female genitalia and the pathological growths developing from embryonal structures. Illustrated by 100 engravings. New York: William Wood & Co. 1901.

The early diagnosis in malignant disease of the uterus has been insisted upon over and again. It has been almost continuously hammered at the general practitioner of medicine, but it has not yet been hammered *into* him; at least it would appear so, from the constant neglect that is observed in this important critical condition. One difficulty has been that it has needed special training to apply the needed methods for minute differential diagnosis in suspected cases. This obstacle to early detection of malignant disease will be removed to a large degree at least by the introduction of Abel's work.

It is not alone malignant disease, such as carcinoma of cervix and uterus, that is important of diagnosis, but endometritis in its various manifestations, polypus, sarcoma and a number of other conditions need early detection and differentiation in order to secure the prompt application of our resources for cure. This author tells us distinctly of technic; that is, how to obtain material and how to use it, how to understand its appearances after preparation, and all in so simple a manner that it makes pathology a delightful study for a physician heretofore unfamiliar with its details.

Abel begins his studies at the vulva and carries them upward along the genital tract to the parovarium, examining each tissue with the utmost care and interpreting carefully all its significances. A chapter, or part, is devoted to embryology of the female genitalia and the pathological growths developing from embryonal structures. This is an important subject in which never before has it been made so clear.

The book is full of splendid illustrations and is a valuable contribution to the study of neoplasms of the genital tract of woman.

A Treatise on Surgery by American Authors. For Students and Practitioners of Medicine and Surgery. Edited by Roswell Park, M. D., Professor of Surgery in the University of Buffalo, N. Y. New (3d) edition. In one royal 8vo volume of 1350 pages, with 692 engravings and 64 full-page plates in colors and monochrome. Philadelphia and New York: Lea Brothers & Co. 1901. [Cloth, $7.00 net; leather, $8.00 net.]

The work before us, now in its third edition, is so well known that but little can be said which has not already appeared in the universally favorable criticisms which have welcomed the previous editions. The present edition is the second since the reduction of the work to one volume and in it certain revisions and changes in the corps of authors become apparent. The previous chapter on surgical gynecology, by Dr. Etheridge, has been replaced by an excellent chapter by Dr. Montgomery A. Crockett, of Buffalo, and the previous chapters on fractures and dislocations have been rewritten by the editor. A short chapter on blood examination and its application to surgery has been separated from the previous chapter on the blood, and now appears over the name of Irving P. Lyon, of Buffalo. The excellent lithographic plate of the blood in normal and diseased conditions which appeared in the second edition of the work, accompanies this chapter.

A marked improvement is found in the book by the removal of some of the less satisfactory of the old illustrations and the introduction of a series of new plates. These are largely photomicrographs, a few selected from other works, but the majority original. Plate 25, a new colored plate, illustrating melanosarcoma, is a great improvement on the old one. The chapter on cysts and tumors shows extensive revision of the text, and 30 new illustrations, mostly printed on plates. It is interesting to note in this chapter the introduction of a paragraph on malignant chorion epithelioma and suprarenal epithelioma or hypernephroma, two important forms of carcinoma, which the recent work of Senn on tumors entirely ignored. The other chapters in this work show more or less extensive revision. It is extremely gratifying to see how ably the editor keeps in touch with all advances in practical surgery and surgical pathology, and it is fair to state that there is no text-book published which so ably sets forth the steady advance of American surgery. Its popularity and success have long been assured, and the new edition, with its many improvements, will be welcomed by many teachers and thousands of students.

CLINICAL HEMATOLOGY. A Practical Guide to the Examination of the Blood with reference to Diagnosis. By JOHN C. DACOSTA, JR., M. D., Assistant Demonstrator of Clinical Medicine, Jefferson Medical College; Hematologist to the German Hospital, etc. Containing 8 full-page colored plates, 3 charts and 48 other illustrations. Octavo, 450 pages. Philadelphia: P. Blakiston's Son & Co. 1901. [Price, $5.00 net.]

The important part played by hematology in the diagnosis of many diseases, makes it incumbent upon the clinician to acquaint himself familiarly with the science; the practising physician, too, may not neglect to equip himself sufficiently to enable him to apply it to the commoner conditions with which he daily comes in contact. In this book will be found the necessary data to guide both classes to a safe conclusion.

Disappointing though it sometimes is and not furnishing pathognomonic evidence of disease, or in not affording the clue of which one is in search, the science of hematology is yet so far advanced as to make it one to be reckoned with by every clinician who would exhaust every known method in his search for essential data that contribute to diagnostic accuracy.

This work represents the present state of this branch of study as evolved in one of the best equipped and adequately conducted laboratories in the country. It also represents the author's larger personal experience in extensive hospital and private practice, and army medical service, hence it may be regarded as the most complete and latest exponent of blood pathology.

One of the most striking and, we might add, essential features is the incomplexness of its descriptive detail, which so simplifies the methods of blood counting, staining and other means of investigation, as to bring it within the comprehension of the beginner; while, at the same time, technic is sufficiently elaborated to meet the conditions of advanced laboratory workers.

Before concluding this important outlining of the principal features of this most extraordinary and invaluable book we feel that a word of tribute is due to its pictorial features. Recognising the importance of correct illustrative work in a book on hematology, the publishers have striven to make the colored plates represent as nearly as possible the subjects as they appear to the eye, and have attained more than the usual amount of success in this direction. With the charts and numerous black and white reproductions the same care in preparation has been exercised. Altogether it is a work to be commended from every viewpoint, and we hope it will be found in the hands at least of every junior physician.

A Text-Book of Medicine. For Students and Practitioners. By Dr. Adolf Strümpell, Professor and Director of the Medical Clinique at the University of Erlangen. Third American edition. Translated by permission from the thirteenth German edition, by Herman F. Vickery, A. B., M. D., Instructor in Clinical Medicine, Harvard University; and Philip Coombs Knapp, A. M., M. D., Clinical Instructor in Diseases of the Nervous System, Harvard University. With editorial notes by Frederick C. Shattuck, A. M., M. D., Jackson Professor of Clinical Medicine, Harvard University. Octavo, pp. 1264, 185 illustrations and 1 plate. New York: D. Appleton & Co. 1901. [Price, cloth, $6.00; leather, $7.00.]

This standard treatise on medicine is a valuable part of the literature of the subject and has been so esteemed since the publication of the first American edition, in 1886. It was again translated and published in this country in 1893, and now we have it for a third time, this one having been translated from the thirteenth German edition. It has also been translated into French, Italian, Russian, modern Greek, Turkish and Japanese, so that it has a pretty world-wide circulation; and of necessity has exercised great influence upon the thought and action of a great number of physicians.

The work has been well prepared for the American profession of medicine, first by the translators, who have made smooth reading English of the text, and next by the editorial notes, inclosed in brackets, that are added here and there. Some of the latter are extensive in scope and afford a most complete elaboration of the subject with which they deal. They are supplied by Professor Shattuck, of Harvard University Medical School.

It is an admitted fact that the Germans have taken a foremost position in investigation relating to internal medicine, and their contributions to its clinical studies have attracted the attention of the professional world. This book contains a conveniently arranged and well digested exposition of the present status of clinical medicine in Europe and America, made so by the combined efforts of author, translators, and editor, all of whom are skilful exponents of the subject. It thus becomes especially adapted to the use both of students and practitioners of medicine, to whom we cordially commend it.

The Practice of Obstetrics. By American authors. Edited by Charles Jewett, M. D., Professor of Obstetrics and Gynecology in Long Island College Hospital, Brooklyn, N. Y. Second edition, revised and enlarged. In one 8vo volume of 775 pages, with 445 engravings in colors and black, and 35 full-page colored plates. Philadelphia and New York: Lea Brothers & Co. 1901. [Price, cloth, $5.00 net; leather, $6.00 net; half-morocco, $6.50 net.]

When the first edition of this splendid work was issued two years and a half ago, we thought and said it was the most complete of the recent obstetric text-books. In the light of further observation and experience we think our judgment was well placed. This new edition exhibits revisions and changes, but the original plan is adhered to and is elaborated. The principal modifications relate to the pathology of pregnancy and obstetric surgery. New light is obtaining almost constantly in pathology, and new fashions are introduced in surgical technic, and the book has not lagged in these respects.

There are eighteen contributors to this volume besides the editor himself, and these represent as many different colleges, thus distributing the authorship among the principal teaching centers. The death of Dr. W. W. Browning necessitated a change in his department, which is now conducted by Dr. A. L. Bristow. Two of the sections formerly in charge of Dr. J. H. Ethridge, upon his death passed into the hands of Dr. M. A. Crockett, of Buffalo, who has rewritten them most admirably. A word should be said relative to the illustrations, many of which are superb, and all of which possess the merit of clearness and accuracy. Tricolor photography plays a satisfactory part, and some of the plates in color are exquisitely done. The treatise is one of the immediate present in everything relating to obstetrics.

---

Modern Obstetrics: General and Operative. By W. A. Newman Dorland, A.M., M.D., Assistant Demonstrator of Obstetrics, University of Pennsylvania; Associate in Gynecology, Philadelphia Polyclinic. Second edition, rewritten and greatly enlarged. Octavo, 797 pages, with 201 illustrations. Philadelphia and London: W. B. Saunders & Co. 1901. [Cloth, $4.00 net.]

This manual of obstetric art has met with deserved approbation, presenting as it does the modern practice of obstetrics, in a clear yet concise form. It is divided most appropriately into two general sections—*physiologic* and *pathologic* obstetrics.

Physiologic obstetrics, on the first part, comprises eight chapters, in the first of which the organs relating to generation and parturition are anatomically considered. In the remaining seven a normal pregnancy is dealt with from conception to the conclusion of lactation. This part of the subject is presented in clear English, in concise verbiage and in scientific form.

The section which considers pathologic obstetrics is necessarily more elaborate, occupying about two-thirds of the book. It discourses upon diseases of the ovum and the fetal appendages, pathologic conditions of the fetus, the pathology of pregnancy, dystocia, pathology of the puerperium, and the pathology of the new-born, in the order named, and presents each in an instructive manner.

We confess to a great liking for this book and sympathise with the author's methods of dealing with the whole subject, differing from him on some minor points of no essential significance. We trust this manual will find its way into the hands of every undergraduate, as it will serve to smooth his pathway in early struggles with a somewhat difficult subject.

---

A MANUAL OF THE PRACTICE OF MEDICINE. By GEORGE ROE LOCKWOOD, M. D., Professor of Practice in the Woman's Medical College of the New York Infirmary; Attending Physician to Bellevue Hospital. Second edition, revised and enlarged. Octavo volume of 847 pages, with 79 illustrations and 20 full-page plates. Philadelphia and London: W. B. Saunders & Co. 1901. [Cloth, $4.00 net.]

The object aimed at by the author in preparing this work was to supply a field intermediate between the comprehensive treatises and the elementary handbooks. How well he has accomplished his purpose is attested by a demand for a second edition in so short a time. His classification is after Osler and his methods of treating his subjects are equally modern.

In the revision there has been rewriting as well as adding to, so that now the book stands quite in the forefront of practice literature. Among the new sections may be mentioned bubonic plague, gastroptosis, gastric analysis and Reichmann's disease. The subject of malaria has been entirely rewritten. The section on diseases of the digestive system has also been largely rewritten, especially the following topics: gastritis, dilatation of the stomach, gastric atony, ulcer of the stomach, gastric neuroses, enteritis and colitis.

It is one of the most complete manuals of medical practice before the profession and readily appeals to students who are not advanced enough to begin the more exhaustive works, and even to junior practitioners of medicine who are not ready for such an outlay. Moreover, it is so concise and clear in its description, and its make-up is so practical, that it becomes for all physicians a valuable book for ready reference; in short, it contains the essentials of internal medicine, stated clearly and in an attractive manner.

---

HUMAN PHYSIOLOGY. Prepared with special reference to Students of Medicine. By JOSEPH HOWARD RAYMOND, A. M., M. D., Professor of Physiology and Hygiene in the Long Island College Hospital, and Director of Physiology in Hoagland Laboratory, New York City. Second edition, entirely rewritten and greatly enlarged. Octavo, 668 pages, 443 illustrations, 12 of them in colors, and 4 full-page lithographic plates. Philadelphia and London: W. B. Saunders & Co. 1901. [Cloth, $3.50 net.]

This useful manual appears in greatly enlarged form and is correspondingly increased in value. The increase to four years in college teaching has made it possible to extend the laboratory courses in this as well as in other branches, hence the author expands his work to meet the changed conditions. The science of physiology, too, has advanced equally with many other subdivisions of medicine, which offers an additional reason for an enlargement of methods of teaching, including working manuals and text-books.

This author has had a teaching experience of more than 25 years and this has served to acquaint him with the needs of students as regards literature. He therefore has prepared this work in accordance with that knowledge, and it appears to be most appropriately constructed. The subdivisions, physiologic chemistry, and the nutritive, nervous, and reproductive functions, that were made in the former work have been retained, but their scope has been very much enlarged. A section on histology has been added, and besides here and there throughout the text enough of this topic is considered to give a better understanding of the physiologic anatomy of the organs under immediate consideration.

There has been much other excellent material added so that, in its enlarged form, the work may justly be classed as a text-book, though never losing entirely its claims as a manual. In other words it is preëminently a book for students, while at the same time it is ample in its scope to serve the needs of the practitioner of medicine—general or special—as a work of reference. We regard it as the best recent contribution to the literature of human physiology.

A MANUAL OF BACTERIOLOGY. By HERBERT U. WILLIAMS, M. D., Professor of Pathology and Bacteriology in the Medical Department of the University of Buffalo. Octavo, pp. 306 With 90 illustrations. Second edition, revised and enlarged. Philadelphia: P. Blakiston's Son & Co. 1901. [Price, $1.50 net.]

This excellent manual will be welcomed in its second edition by students of bacteriology as a most helpful guide in laboratory work. Dr. Williams is a master of his subject, a careful teacher, and a thorough delineator of laboratory technique. He has put as much of the topic into book form as will prove useful to the student in his earlier researches, and enough to serve as a substantial aid to the medical practitioner who desires to pursue bacteriologic investigations.

The book is somewhat larger than the first edition, rendered so by necessary additions in the revision, but it is still of convenient size for the purposes it is designed to meet. The chapter on surgical disinfection by Dr. Chauncey P. Smith is of great practical importance; and one on disinfectants and antiseptics by Dr. Thomas B. Carpenter, is deserving of careful study. The student and junior physician should master both of these subjects. There is too much socalled disinfection and not enough of the real thing—too much disinfection that does not disinfect. These chapters will serve to correct this evil.

Again, we commend this manual to the beginner and to the physician who would pursue bacteriologic studies after entering practice.

---

INTERNATIONAL CLINICS. A Quarterly of Clinical Lectures and Especially Prepared Articles on Medicine, Neurology. Surgery, Therapeutics, Obstetrics, Pediatrics, Pathology, Dermatology, Diseases of the Eye, Ear, Nose and Throat, etc Edited by HENRY W. CATTELL, A. M., M. D., of Philadelphia, with the collaboration of John B. Murphy, M. D., of Chicago; Alexander D Blackader, M. D., of Montreal; H. C. Wood, M. D., of Philadelphia; T. M. Rotch, M. D., of Boston; E. Landolt, M. D., of Paris; Thomas G. Morton, M. D., and Charles H Reed, M. D., of Philadelphia; J. W. Ballantyne, M. D., of Edinburgh, and John Harold, M. D., of London. Volume IV. Eleventh series. Philadelphia: J. B. Lippincott Co. 1902 [Price, cloth, $2.00 each; half-leather, $2.25 each.]

The subjects treated in this number of the clinics are therapeutics, medicine, neurology, surgery, pediatrics, dermatology, and there is a special article on the methods of keeping case records in private practice. Among the contributors are A. Jacobi, Horatio Wood, Jr., Charles H. Burnett, Norman Bridge, Wm. C. Krauss, Charles H. Chetwood, Nicholas Senn, G. Frank Lydston, John Madison Taylor, and William S. Gottheil, at home; and L. Brocq, Albert Mathieu, Pierre Delbet, Sir Dyce

Duckworth, J. Mitchell Bruce, G. Marinesco, P. Quénu, Thomas Jonnesco and James Cantlie, abroad. The special article is written jointly by J. P. Crozer Griffith, Judson Daland, J. K. Mitchell, John H. Musser and Alfred Stengel. The foregoing will convey an idea of the value of the book, the authors being men of recognised standing.

---

THE HISTORY OF MEDICINE IN THE UNITED STATES. A collection of facts and documents relating to the history of medical science in this country, from the earliest English colonisation to the year 1800. With a supplementary chapter on the discovery of anesthesia. BY FRANCIS RANDOLPH PACKARD, M. D. Octavo, pp. 542. Illustrated. Philadelphia: J. B. Lippincott Co. 1901. [Price, $4.00.]

Such a book as this is of inestimable value to physicians; it enables them to link the present with the past in their readings and keeps them in touch with the fathers of medicine in this country, men whose sturdy example and wholesome influence laid the foundation for an enduring and honorable profession.

The colonial history is of great interest and it is a pity that the available material is so meager. The history of medical societies founded previous to 1800, is of very great value. It necessarily tells of first things and in this it is of importance. The discovery of anesthesia will be read with much interest by all who feel doubt as to its discoverer and demonstrator.

The entire book is prepared in an attractive way; its subjects are presented in entertaining form. The author has taken great pains to verify his facts and data, so, without doubt, it is by far the best history of the early features of medicine in the United States that has ever been prepared.

---

THE TECHNIQUE OF SURGICAL GYNECOLOGY. Devoted exclusively to a description of the technique of gynecological operations. By AUSTIN H. GOELET, M. D., Professor of Gynecology in the New York School of Clinical Medicine. Duodecimo, pp. 340. Illustrated. New York: International Journal of Surgery Co. 1901.

The description of operative technic and preparation for operations is quite clear and given in sufficient detail in this work for all needful purposes.

The author has had considerable experience, and it is to be expected that he knows how to prepare patients, surgery, and assistants for operation, and to how the operation should be conducted. The work is limited to this field and in this respect is unique.

The book is not padded with irrelevant material, but goes straight at its task and stops when it is finished. There are a few typographical errors that will no doubt receive attention in a later edition. If there is any criticism to offer, we should incline to regard the author as over fond of a multiplicity of instruments, and of attaching his own name to a number of comparatively unimportant inventions or modifications of those instruments devised by others. However, this is of small consequence as compared with the general usefulness of the book, which we regard as a fixed and determined fact.

---

DOSE-BOOK AND MANUAL OF PRESCRIPTION WRITING: with a List of Official Drugs and Preparations, and the more important Newer Remedies. By E. Q. THORNTON, M. D., Demonstrator of Therapeutics, Jefferson Medical College, Philadelphia. Second edition, revised and enlarged. Octavo, 362 pages, illustrated. Philadelphia and London: W. B. Saunders Co. 1901. [Bound in flexible leather, $2.00 net.]

This is a convenient book for reference and belongs to a class that seems to be quite popular, especially with junior practitioners of medicine. Its chief value, however, is in the instruction it imparts to the student in regard to writing prescriptions. A correct start should be made in this respect, and then it is apt to last through life. It is so important to observe grammatical accuracy in this work that no amount of time spent upon acquiring the requisite knowledge should be counted as wasted or as devoted to trivialities. The dosage part of the book, though of less consequence, is still of importance and has been well prepared. It contains much information not easily accessible and is commended for its scholarly effort to impart useful knowledge to the ambitious searcher.

---

REPORT of the Commissioner of Education for the Year 1899-1900. Two volumes. Washington: Government Printing Office. 1901.

This report is of interest to all educators, but some parts of it will attract physicians in particular. Of these we may mention a paper, by Professor W. O. Atwater, of Wesleyan University, Middletown, Conn., read at the Chicago meeting of the department of superintendence, 1900, entitled, Alcohol, Physiology and Superintendence; and of special importance is what is said in it relating to what should be taught in the schools about alcohol. It takes a calm and scientific view of the subject. Another paper of interest is the address by Judge Baldwin, of Connecticut, before the legal education section of the American Bar Association, 1898, on the Readjustment of the Collegiate to the Professional Course. There are, of course, many other important papers and statistics in these two volumes which will bear patient examination. It is a matter of regret that these reports are not printed better and bound stronger.

THE SURGICAL TREATMENT OF DISFIGUREMENTS AND DEFORMITIES OF THE FACE. By JOHN B. ROBERTS, A. M., M. D., Professor of Surgery in the Philadelphia Polyclinic; Surgeon to the Methodist Hospital. Duodecimo, pp. 72. Second edition, with a chapter on the Reconstruction of Syphilitic Noses. Sixty-two figures. Philadelphia: The Philadelphia Medical Publishing Co. 1901.

The class of deformities discussed in this brochure are among the unsightliest that afflict the human race. The author has had much experience in dealing with them and is entitled to great credit in the skilful surgery which he employs in repairing such disfigurements. The illustrations are varied and graphic, making plain the author's methods, and impressing the reader with their perfection. The chapter on reconstruction of syphilitic noses is a valuable addition to this edition.

TRANSACTIONS of the Medical Association of the State of Alabama (The State Board of Health). Annual meeting, held at Selma, April 16–19, 1901. G. P. WALLER, M. D., Secretary.

This book compares favorably with the transactions of the medical societies of larger states, containing many valuable papers of scientific interest. The annual address of the president, called here his "message," is the first paper, and then follow "reports" of the senior and junior vice-presidents. These are some of the unique features of the organisation. There are others that are distinctive, but we need not refer to them in detail. The volume is well edited and is printed in excellent form. The medical profession of Alabama may well be proud of its status; it is well organised and its esprit de corps is enviable.

---

TRANSACTIONS of the State Medical Association of Texas. Thirty-third annual session, held at Galveston, Texas, April 23, 24, 25 and 26, 1901. H. A. WEST, M. D., Secretary.

This progressive society again presents its annual volume of transactions in attractive form. It is replete with interesting scientific papers and valuable reports of committees. Some of these are discussed but the discussions, generally speaking, are rather meager. It is a book, however, that reflects credit upon the association and upon the distinguished secretary who edited it.

